# Social media for palliative and end-of-life care research: a systematic review

**DOI:** 10.1136/spcare-2023-004579

**Published:** 2024-04-09

**Authors:** Yijun Wang, Jonathan Koffman, Wei Gao, Yuxin Zhou, Emeka Chukwusa, Vasa Curcin

**Affiliations:** 1 Department of Population Health Sciences, King's College London, London, UK; 2 Wolfson Palliative Care Research Centre, Hull York Medical School, Hull, UK; 3 Epidemiology & Health Statistics, Nanchang University, Nanchang, China; 4 Cicely Saunders Institute of Palliative Care, King's College London, London, UK

**Keywords:** Ethics, Terminal care, Education and training, Communication, Supportive care

## Abstract

**Background:**

Social media with real-time content and a wide-reaching user network opens up more possibilities for palliative and end-of-life care (PEoLC) researchers who have begun to embrace it as a complementary research tool. This review aims to identify the uses of social media in PEoLC studies and to examine the ethical considerations and data collection approaches raised by this research approach.

**Methods:**

Nine online databases were searched for PEoLC research using social media published before December 2022. Thematic analysis and narrative synthesis approach were used to categorise social media applications.

**Results:**

21 studies were included. 16 studies used social media to conduct secondary analysis and five studies used social media as a platform for information sharing. Ethical considerations relevant to social media studies varied while 15 studies discussed ethical considerations, only 6 studies obtained ethical approval and 5 studies confirmed participant consent. Among studies that used social media data, most of them manually collected social media data, and other studies relied on Twitter application programming interface or third-party analytical tools. A total of 1 520 329 posts, 325 videos and 33 articles related to PEoLC from 2008 to 2022 were collected and analysed.

**Conclusions:**

Social media has emerged as a promising complementary research tool with demonstrated feasibility in various applications. However, we identified the absence of standardised ethical handling and data collection approaches which pose an ongoing challenge. We provided practical recommendations to bridge these pressing gaps for researchers wishing to use social media in future PEoLC-related studies.

**WHAT IS ALREADY KNOWN**

- Social media is widely used in healthcare research and most recently during COVID-19. Its uses in healthcare research have been summarised and criticised. Palliative and end-of-life care researchers have gradually applied social media in their research. However, concerns were raised around ethical issues (eg, privacy and anonymity) and data quality when using social media as a research tool. It is necessary to systematically review its uses in this research field and examine its ethical handling and data collection to inform future researchers.

**WHAT ARE THE NEW FINDINGS**

- This review identified the increasing use of social media data to conduct secondary analysis for palliative and end-of-life care research. However, we identified inconsistent consideration of ethical issues relevant to its use and non-standardised approaches to data collection. This highlighted concerns regarding the ethical principles associated with this method of data collection and analysis as well as its quality.

**WHAT IS THEIR SIGNIFICANCE**

- This review highlighted social media’s value in contributing to palliative and end-of-life care-related studies both as a means of data collection and promoting research. Based on our findings, we developed practical recommendations on the use of social medial research in palliative and end-of-life care studies, including flexible ethical review criteria, ethical risk mitigation strategies, data validation and non-English platform research. Our pragmatic recommendations serve as a reference for future researchers to help them conduct more robust and rigorous palliative social media research.

## Introduction

Social media is defined as a collection of internet-based applications to facilitate the creation and exchange of user-generated content.^1^ A classification scheme has been developed to delineate the different types of social media,^1^ which includes collaborative projects, for example, Wiki; blogs or microblogs, for example, Twitter; content communities, for example, YouTube; social networking sites that include Facebook; and virtual worlds, for example, Second Life. Globally, the number of social media users has increased dramatically since its inception to approximately 4.74 billion in January 2022.^2^ This represents 58.4% of the world’s population.^2^ The healthcare field has embraced social media as a useful tool to access and share information. In 2018, 67% of American healthcare information seekers reported accessing information on social media.^3^ Social media has also increasingly been used in healthcare research to provide health information, answer health questions, facilitate health dialogue, collect patient data, reduce stigma, and provide online education and consultations.^4^

Palliative and end-of-life care is an essential component of a healthcare system.^5^ The increasing engagement in social media of palliative and end-of-life care stakeholders creates a ready platform for its application in palliative and end-of-life care research.^6^ Eng *et al*’s study^7^ identified that among 371 cancer survivors, 74% used the internet and 39% specifically used social media for accessing cancer care information. Other studies have observed that social media is frequently used by patients with cancer to connect with peers and develop stronger bonds with family members.^8^ In the 2018 National Cancer Institute’s Health Information National Trend survey, respondents ranked online sources, including the internet and social media, as their second choice for seeking palliative care knowledge, after that from healthcare providers.^9^

A growing body of literature has used social media in palliative and end-of-life care research.^10–13^ A recent study showed that social media platforms provided a time-efficient and cost-effective method for recruiting paediatric oncology patients for palliative care research.^10^ In addition, social media platforms have been increasingly used to conduct secondary data analysis to understand barriers to patients accessing palliative care,^11^ evaluate educational online resources for the public^12^ and examine determinants of social behaviours and beliefs towards palliative and end-of-life care.^13^ Advances in natural language processing technologies have enabled researchers to extract useful information from unstructured social media data such as demographic features, views and emotional sentiment of participants which provide valuable insights.^13–15^

Despite promising benefits, when used as a tool for research, social media is open to criticism. While there is an increasing number of studies that have focused on how to conduct social media research, few studies have examined what constitutes high-quality and ethically responsible social media research. Roland *et al* 16 and Teague *et al* 17 attempted to develop guidelines for social media studies, however, they only focused on specific domains, for example, mental health and emergency care. Kaushal *et al* 18 are currently attempting to construct a more general guideline, but it is still ongoing. Standardised guidelines for social media research are therefore still scarce. Despite this, common concerns have been identified and included in existing social media research guidelines, for example, ethical issues and data quality within the healthcare domain.^17 19–21^ Although standardised criteria are currently absent, it is suggested that healthcare researchers should adopt a cautious approach to ethical issues and ensure data accuracy and reliability when using social media.^19–21^ Therefore, there is a clear imperative to review and examine these two centrals concerns when conducting palliative and end-life care studies using social media.

It has been claimed that the introduction of social media to palliative and end-of-life care research presents ethical challenges to researchers that include privacy, anonymity and content ownership.^6^ When it comes to the context of palliative and end-of-life care, ethical considerations may be amplified due to the potential vulnerability of participants^22 23^ and the personal and sensitive information shared on social media. This has potential legal implications and ramifications for General Data Protection Regulation (GDPR), a European Union regulation on information privacy in the European Union and the European Economic Area. An ethical guidance^20^ to inform the use of potentially sensitive social media data suggests researchers must either (a) paraphrase all data which is republished in research outputs; (b) seek informed consent from each person or (c) consider using a more traditional research approach. However, the extent to which ethical considerations have been addressed in existing palliative care research remains ambiguous.

Furthermore, if we are to develop a robust evidence base to inform the delivery of palliative and end-of-life care, high-quality data are critical.^24^ However, the quality of social media data has been criticised because of apparent inaccuracies and biases.^21^ Consequently, a focus on data collection and verification specific to social media in palliative and end-of-life care research is essential. A complete social media data collection and verification should contain three steps: develop, apply and validate.^25^ This systematic review, therefore, aimed to (1) identify and appraise different applications of social media in palliative and end-of-life care studies, (2) examine the ethical considerations when using this research approach, (3) examine data collection and verification approaches when using this research approach and (4) make recommendations for researchers who wish to integrate social media in their future research.

## Methods

### Study design

This systematic review followed the Preferred Reporting Items for Systematic Reviews and Meta-Analyses 2020 guidelines.^26^ The study protocol was registered with the International Prospective Register of Systematic Reviews (CRD42021262026).^27^

### Search strategy

A two-stage search strategy was applied including a preliminary search to identify search terms and a full search to identify related literature. First, a preliminary search was conducted to explore search terms related to the review questions. The selection of search terms was informed by key terms and associated controlled terms used in relevant palliative and end-of-life care^28–30^ or social media review papers.^31–34^ Palliative care research experts were also consulted to further identify appropriate search terms. The final search terms are presented in table 1.

**Table 1:** Search terms

Concept	Search terms
Social media	“social media” OR “social web” OR “social network” OR “web 2.0” OR “web2” OR “web-based
	“Twitter” OR “tweet*” OR “YouTube” OR “LinkedIn” OR “Instagram” OR “Reddit” OR “Weibo” OR “WeChat” OR “online forum*” OR “online community” OR “Pinterest” OR “Tumblr” OR “TikTok” OR “PatientsLikeMe” OR “blog”
Palliative and end-of-life care	“palliative*” OR “hospice” OR “end of life” OR “EoL*” OR “PEoL*” OR “terminal care” OR “terminal ill*” OR “advance care” OR “Marie Curie nurse” OR “Macmillan nurse” OR “comfort care” OR “supportive care” OR “bereavement care” OR “respite care” OR “pain management” OR “pain control” OR “symptom management”

In the second stage, a full search was conducted to identify related papers among seven health-related electronic databases: Medline, Embase, PsycINFO, Global Health, Health Management Information Consortium, Web of Science (Core Collection), Chinese National Knowledge Infrastructure (CNKI) and two grey literature databases: OpenGrey and CareSearch. The detailed search strategy for each database was listed in online supplemental file 1. The search was initiated on 9 June 2020, with the most recent update conducted on 30 December 2022.

10.1136/spcare-2023-004579.supp1 Supplementary data

### Eligibility criteria

Palliative and end-of-life care research can broadly be defined as studies that attempt to investigate the physical, psychosocial, spiritual and existential needs of patients living with a life-threatening illness and their families, and the evaluation of the effectiveness and cost-effectiveness of interventions, across all settings, to address specific patient-centred concerns and maximise the quality of life for these individuals and their families.^35^ Social media was defined as platforms encompassing collaborative projects, blogs or microblogs, content communities, social networking sites, and virtual worlds according to Kaplan and Haenlein’s classification scheme.^1^ Studies meeting the following inclusion criteria were included: (1) peer-reviewed journal articles with a focus on ‘palliative and end-of-life care research’ and ‘social media’; (2) where methodology and results were provided and (3) where social media was used to obtain at least one part of the results. Since our review aimed to have a comprehensive and up-to-date understanding of social media applications in palliative and end-of-life care research, there were no restrictions on population, language, study design and publication year to ensure the comprehensiveness of the review. Literature reviews, conference abstracts and letters were eventually excluded since they provided limited information about ethical and methodological issues. This is deviated from the original protocol because we found that during the course of the review, researchers have major concerns about ethical issues and data quality when using social media.

### Data selection

Papers from different databases were merged and imported into the EndNote V.X9 to facilitate the identification of duplicates and to screen publications. Two reviewers (YW and EC) independently applied inclusion and exclusion criteria to screen titles and abstracts of all papers and then the full text of the remaining papers. Discrepancies were resolved by a third reviewer (WG) through discussion until a consensus was reached.

### Quality assessment

Since we included qualitative, quantitative and mixed-method studies in this review we have adopted the QualSyst tool^36^ to evaluate the quality of the included studies. This tool is a good fit when evaluating research papers encompassing a variety of different research approaches.^37^ Furthermore, it has been used in previous systematic reviews examining emerging research tools.^38 39^ For assessing the quality of qualitative studies, 10 standard criteria (research question, study design, context, theoretical framework, sampling strategy, data collection method, data analysis, verification procedure, conclusion and reflexivity of the account) were scored; while for quantitative studies, 14 criteria (research question, study design, method of subject selection, subject characteristics, outcome measures, sample size, analytical methods, estimate of variance, confounding, results, conclusions and, in cases of intervention studies, to the allocation and blinding) were scrutinised. For mixed-method studies, we applied QualSyst tools to their qualitative and quantitative components, respectively, and then calculated the mean score. The quality score does not state anything about the quality of social media uses in included studies since social media research has its own checklist (although not standardised yet), but only indicates the extent to which the design, conduct and analyses attempted to minimise errors and biases. Based on previous studies using QualSyst,^40^ a summary score was used to assess quality where scores of >80% were judged as ‘strong’, 71%–80% as ‘good’, 51%–70% as ‘adequate’ and <51% as ‘limited’. Two reviewers (YW and EC) performed the quality assessment independently. Any discrepancy was resolved by a third reviewer (JK) through discussion until a consensus was reached.

### Data extraction

Data from studies that met the inclusion criteria were extracted to an Excel spreadsheet. Information extracted included basic information (title, authors, publication year, publication type, country) and information related to our review questions (study design, study objectives, social media platform, main application, ethical considerations). To further characterise data collection approaches, specified metrics were extracted from empirical studies using social media data including data extraction method, searching keywords, start and end date, and number of posts/videos collected. YW performed the data extraction independently.

### Data analysis and data synthesis

We adopted thematic and narrative synthesis methods to categorise social media applications.^41^ This comprised three stages: ‘line-by-line’ coding, developing descriptive themes by grouping the coded results into a hierarchical tree structure and generating analytical themes by answering review questions.^41^ Analytical themes represent a stage of interpretation from the review question’s perspective and reviewers have to go beyond the original content and generate reasonable and logical hypotheses.^41^ In our review, social media applications were categorised according to this process and analytical themes were inferred by considering how social media supported palliative and end-of-life care research.

We categorised social media applications under two distinct social media approaches: social media as a secondary data source and social media as a platform for sharing information.^42^ These two approaches were proposed for scrutinising the use of social media in health research.^42^ Secondary data refers to social media data that was already available on platforms before a study was conducted and provides a starting point for research or helps support findings.^43^

We synthesised data collection and verification approaches based on a widely used social media data collection framework,^25^ where social media data collection approaches are defined as approaches for (1) developing a search filter, (2) applying the search filter to retrieve and collect data and (3) assessing the search filter. A search filter is necessary to obtain relevant data for the research topic when searching on social media platforms, which includes a set of keywords integrated with search rules. Data collection approaches were summarised from these three steps.

## Results

### Search results and study characteristics

**Figure 1:**
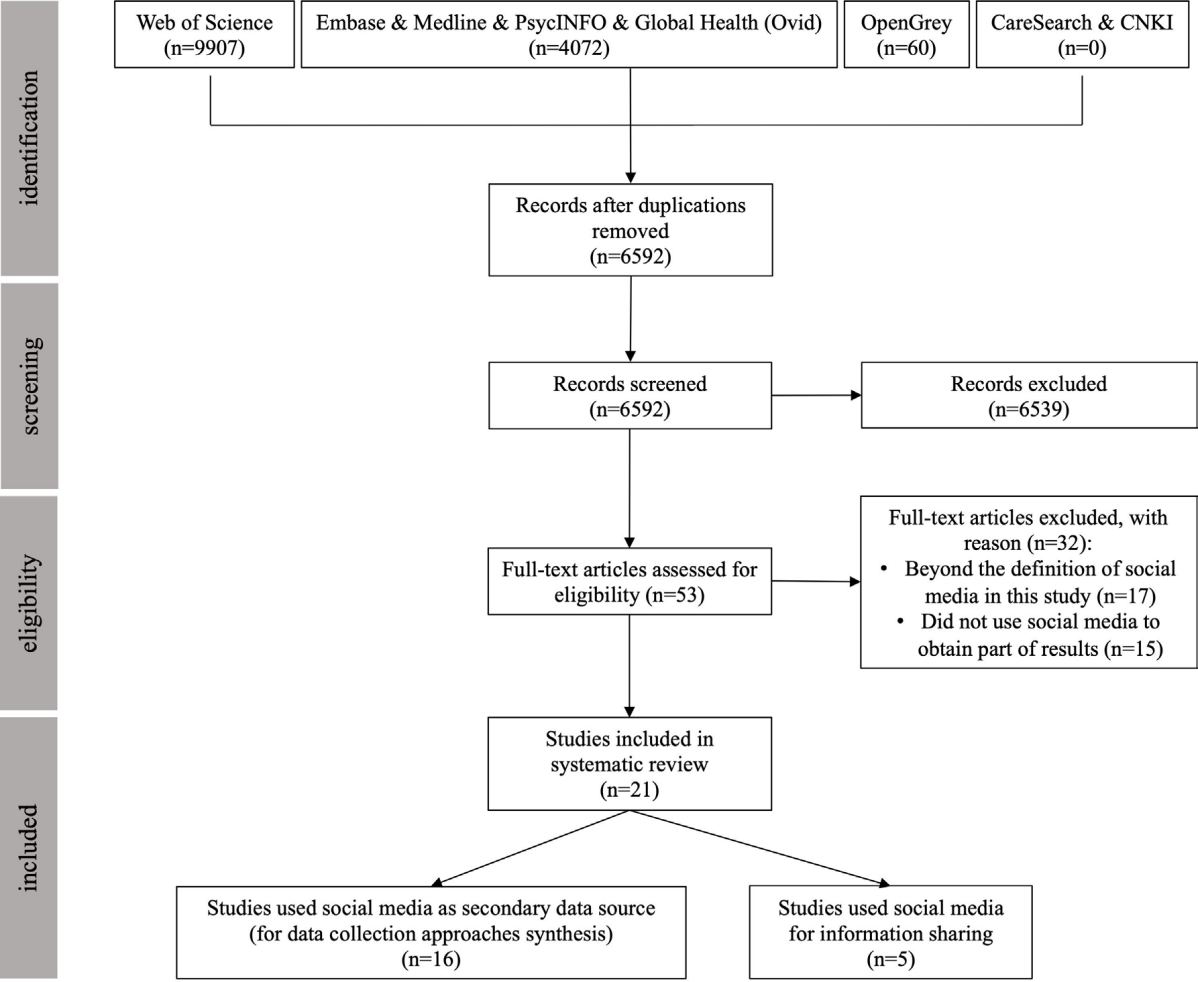
Flow chart of study selection based on the guidelines of PRISMA. PRISMA, Preferred Reporting Items for Systematic Reviews and Meta-Analyses.

As of 30 December 2022, 6592 papers were screened of which 53 papers went to full screening leading to 21 papers that were included in this review (figure 1). The quality of the articles was appraised as ‘strong’,^15 44–48^ ‘good’,^10 12 49–56^ ‘adequate’^13 57–59^ or ‘limited’.^60^ Quality scores and other characteristics of the included studies are presented in table 2. Overall, there was an increasing trend over time in the number of published papers using social media for palliative and end-of-life care research. From 2017, the average annual number of publications was higher than in previous years. 76% (n=16) of included studies were from the USA followed by the UK contributing to 14% (n=3) of included studies. The remaining studies were from Australia (n=1) and Bangladesh (n=1).

**Table 2:** Characteristics of included studies

Reference	Study design (quality assessment, %)	Platform	Social media approach	Social media application	Ethical considerations	Data collection		
						Data extraction tool	Searching key terms	Number of posts/videos/articles
Salmi *et al* (2020)^44^	Qualitative study (85)	Twitter	As a secondary data source	To engage stakeholders to understand their experiences or thoughts	‘Non-Human Subjects Research’ determined by the Institution Review Board.	Symplur	#BTSM, #HPM	772 tweets
Padmanabhan *et al* (2021)^45^	Cross-sectional study (85)	Twitter	As a secondary data source	To engage stakeholders to understand their experiences or thoughts and and to investigate surveillance on the frequency, trend and features of public conversation	Exempt from ethical review determined by the Institution Review Board.	Symplur Signals	#palliativeCare	182 661 tweets
Cutshall *et al* (2020)^57^	Qualitative methods (65)	Twitter	As a secondary data source	To engage stakeholders to understand their experiences or thoughts	Not Human Subjects Research determined by the institutional review board.	Manually collected	#BTSM	536 tweets
Taylor and Pagliari (2018)^49^	Qualitative study (80)	Twitter	As a secondary data source	To engage stakeholders to understand their experiences or thoughts	Ethics approval was provided by the Institutional Review Board. Written agreement was sought and obtained.	Manually collected	@GrangerKate	550 tweets
Claudio *et al* (2018)^50^	Cross-sectional study (75)	YouTube, Facebook and Twitter	As a secondary data source	To explore the quality and features of online resources	Exempt from ethical review determined by the institutional review board.	Manually collected	“palliative care” (YouTube), “#palliativecare”, “#pallcare”, and “#palcare” (Twitter)	25 tweets, 25 Facebook, 10 videos
Mitchell *et al* (2017)^58^	Mix-method study (70)	YouTube	As a secondary data source	To explore the quality and features of online resources	Not reported	Manually collected	‘Advance Care Directive’ OR ‘ACP’ OR ‘Advance Care Plan’	42 videos
Nwosu *et al* (2015)^15^	Retrospective analysis (quantitative study) (83)	Twitter	As a secondary data source	To investigate surveillance on the frequency, trend and features of public conversation	Non-human subjects, therefore ethics committee approval was not deemed to be necessary.	TopsyPro	#hpm #hpmglobal #eolc #Hospice care Liverpool care pathway	683 500 tweets
Zhao *et al* (2020)^13^	Quantitative study (64)	Twitter	As a secondary data source	To investigate surveillance on the frequency, trend and features of public conversation	Not reported	Twitter API	“palliative care” and “palliative medicine” etc.	371 880 tweets
Buis (2008)^46^	Qualitative content analysis (90)	Yahoo!	As a secondary data source	To explore the quality and features of online resources	Was approved by a university institutional review board and adhered to guidelines for ethical research.	Manually collected	hospice	443 posts
Singh *et al* (2021)^51^	Qualitative content analysis (75)	Twitter	As a secondary data source	To engage stakeholders to understand their experiences or thoughts	Ethical approval was obtained from the institutional ethics committee. Consent was waived.	Manually collected	#pallicovid and #COVID-19, palliative care OR COVID-19 OR death OR dying	33 online articles and blogs
Guidry and Benotsch (2019)^52^	Mix-method study (80)	Pinterest	As a secondary data source	To investigate surveillance on the frequency, trend and features of public conversation	Institutional review board approval was not required.	Manually collected	Chronic Pain	502 pins
Patel *et al* (2022)^53^	Mixed-method study (75)	Twitter	As a secondary data source	To investigate surveillance on the frequency, trend and features of public conversation	Institutional review board approval was not required.	Twitter API	“DNR”, “DNI”,“advance directives,” “ECMO,” “CPR,” “dialysis,” and “ventilation,” “advance directives” etc.	202 585 tweets about LSIs and 67 162 tweets about ACP
Easwar *et al* (2022)^54^	Mixed-method study (74)	TikTok	As a secondary data source	To explore the quality and features of online resources	Not reported	Manually collected	‘palliative.’	146 videos
Lattimer *et al* (2022)^59^	Mixed-method study (65)	Twitter	As a secondary data source	To investigate surveillance on the frequency, trend and features of public conversation	Not reported	Twitter API	#nhdd, #advancecareplan, and #goalsofcare, healthcare decision day, advance care plan, goals of care.	9713 tweets
Liu *et al* (2019)^12^	Review (73)	YouTube	As a secondary data source	To explore the quality and features of online resources	Institutional review board approval was not required.	Manually collected	palliative care	84 videos
Wittenberg-Lyle *et al* (2014)^55^	Review (75)	YouTube	As a secondary data source	To explore the quality and features of online resources	Not reported	Manually collected	pain and cancer; pain and hospice; pain and PC palliative care	43 videos
Levy *et al* (2019)^47^	Experimental study (82)	Pixstori	As an information sharing platform	To deliver intervention	Was approved by the institutional review board. Written consent and media waiver were obtained.	Not applicable	Not applicable	Not applicable
Taubert *et al* (2018)^48^	Mixed-method design (88)	YouTube, Facebook and Twitter	As an information sharing platform	For promotion, education and training	No ethics approval was required. Consent and agreement were obtained.	Not applicable	Not applicable	Not applicable
Akard *et al* (2015)^10^	Survey (quantitative study) (79)	Facebook	As an information sharing platform	To enhance recruitment opportunities	Was approved by the institutional review board. Consent was obtained online.	Not applicable	Not applicable	Not applicable
Biswas *et al* (2021)^61^	Cross-sectional study (75)	Not reported	As an information sharing platform	To enhance recruitment opportunities	Was approved by the ethical review committee. Informed consent was obtained.	Not applicable	Not applicable	Not applicable
Beringer *et al* (2017)^60^	Qualitative methodology (45)	Self-built platform	As an information sharing platform	For promotion, education and training	Not reported	Not applicable	Not applicable	Not applicable

API, application programming interface.

The proportion of publications using different social media platforms is presented in table 2. From 2015 to 2022, Twitter was the most commonly used (n=11) social media platform. The second most frequently used platform was YouTube (n=5) followed by Facebook (n=3). The oldest (2008) platform identified was Yahoo! (n=1) and the newest was TikTok (n=1). Picture-sharing platforms such as Pinterest (n=1) were also represented. While most studies made use of popular social media platforms, one study^60^ made use of its dedicated online community serving older LGBTQ+ individuals regarding end-of-life planning. One paper used more than one platform in their studies.^50^

### Social media applications in palliative and end-of-life care research

Based on the taxonomy,^42^ we identified three applications using social media as a secondary source of data which included (1) exploring the quality and features of online resources; (2) engaging with stakeholders to understand their experiences and thoughts; (3) investigating surveillance of the frequency, trends and features of public conversations. In addition, we identified three applications using social media as a platform for sharing information which included (1) delivering intervention; (2) enhancing recruitment opportunities and (3) for promotion, education and training. The number of studies in each classification scheme is presented in table 3.

**Table 3:** Social media application in palliative and end-of-life care research

Social media approaches	Application	No of studies
As a secondary data source (n=16)	To explore the quality and features of online resources	6^12 46 50 54 55 58^
	To engage stakeholders to understand their experiences or thoughts	5^44 45 49 51 57^
	To investigate surveillance on the frequency, trend and features of public conversation	6^13 15 45 52 53 59^
As a platform for information sharing (n=5)	To deliver intervention	1^47^
	To enhance recruitment opportunities	2^10 61^
	For promotion, education and training	2^48 60^

#### Social media as a secondary data source (n=16)

16 studies were characterised as secondary data analysis studies. Specifically, these studies have been categorised into three groups. Six studies^12 46 50 54 55 58^ explored the quality and features of online resources using social media. Three of them^12 55 58^ summarised video resources on YouTube and one research study^54^ explored short-form videos on TikTok. One study^50^ compared the resources available on YouTube, Facebook and Twitter and identified that YouTube was able to provide valuable insights into examining palliative care resources. Some of these studies^12 50^ attempted to identify how palliative care was portrayed in social media videos and they found most resources were consistent with the current definition of palliative care. Two studies^54 58^ attempted to explore the relationship between resource features, for example, author characteristics and content type and public engagement (eg, the number of views, ‘likes’ and ‘forwards’) to inform the future development of online resources. One study^46^ described the different types of social support in a hospice online community and found emotional support was higher than informational support.

Five studies^44 45 49 51 57^ used social media to engage stakeholders to understand their experiences or insights about palliative and end-of-life care. One study^45^ focused on self-identified informal caregivers and summarised their tweets to explore their experiences of palliative care. Most recently, Singh *et al* 51 explored health professionals’ Twitter articles and blogs to ascertain their views on the role of palliative care during the COVID-19 pandemic. One study^49^ collected 550 tweets from a single cancer patient to provide a detailed perspective of her end-of-life experiences. Two studies used Twitter chatter data to understand the quality of life needs^44^ and advance care planning experiences^57^ of patients living with brain tumours, and the perspectives of their caregivers, healthcare professionals and organisations.

Last, six studies^13 15 45 52 53 59^ investigated surveillance of public conversation about the frequency, trend and features on social media. These studies traced public discussion on Twitter or Pinterest from 2011 to 2021 on a range of palliative and end-of-life care topics including palliative care,^13 15 45^ chronic pain,^52^ life-sustaining interventions^53^ and advance directives.^59^ Frequency surveillance,^15 45^ content analysis,^13 45 52 53 59^ user demographic feature prediction,^13 53^ network analysis^45^ and sentiment analysis^15 45 53^ were applied in these studies to understand the discussion frequency, popular topics, user’s gender or age, information dissemination network and public sentiment (eg, positive or negative sentiment) towards palliative and end-of-life care social media discussion.

#### Social media as a platform for information sharing (n=5)

Five studies^10 47 48 60 61^ were identified as using social media as a platform for information dissemination. The applications of social media as a platform have been categorised into the following three groups. First, one study^47^ used social media to deliver palliative care intervention. This study^47^ used social media to deliver an intervention for paediatric palliative caregivers via the ‘Photographs of Meaning Programme’. Specifically, participants were asked to post photo narratives on social media. Second, social media was used to enhance recruitment opportunities in two studies.^10 61^ Akard *et al*’s empirical studies tried to validate the cost efficiency of social media in recruitment which used Facebook to recruit paediatric cancer patients.^10^ Biswas *et al* 61 recruited clinicians through social media to understand their knowledge about palliative care. Last, two studies^48 60^ examined the place of social media for promotion, education and training for palliative and end-of-life care. Twitter and YouTube were used in one study^48^ to raise public awareness of palliative and end-of-life care by disseminating self-made videos to improve communication for do not attempt cardiopulmonary resuscitation decision. This study reported more than 100 000 hits over 6 months. Another study^60^ shared end-of-life information for older LGBTQ+ adults through a self-developed supportive online community. However, the decreasing engagement of this forum was reported, possibly attributed to the lack of security and privacy in the online environment.

### Ethical considerations

A total of 15 out of 21 studies discussed ethical considerations associated with the use of social media in palliative and end-of-life care research. Specifically, we identified that when using social media as a platform for information sharing researchers generally considered that ethical approval and participant consent were warranted. All five social media studies^10 47 48 56 61^ using social media as a platform reported ethical considerations, with four^10 47 48 56^ obtaining informed consent and three^10 47 61^ obtaining ethical approval. In contrast, our findings revealed that when using social media as a secondary data source, researchers often perceived that seeking ethical approval and participant consent was sometimes unnecessary. 11 out of 16 studies^12 15 44–46 49–53 57^ reported their ethical considerations for using social media data. Specifically, eight studies^12 15 44 45 50 52 53 57^ stated ethical approval was exempt because of the public availability of social media data, and therefore, no consent was obtained. Two studies^46 51^ obtained ethical approval but the need to obtain consent was waived. One study^49^ obtained both ethical approval with associated consent from the participant and her family. The results indicated that the distribution of ethical considerations of two social media applications was highly variable (see table 4).

**Table 4:** Ethical considerations status among two social media applications

Ethical considerations	The number of studies using social media as a secondary data source	The number of studies using social media as a platform for information sharing
No discussion	5	1
Exempt from ethical approval	8	1
Obtained ethical approval	3	3
Obtained informed consent	1	4
Total	16	5

### Social media data collection approaches

A total of 16 studies^12 13 15 44–46 49–55 57–59^ used secondary social media data. Here, we synthesised the data collection approaches of these studies.

When developing a search filter, identified studies used various keywords related to their research topics. Six studies^12 13 45 50 51 54^ focused on palliative and hospice-care-related topics on social media and used keywords including #PalliativeCare, #hpm, #eolc, #Hospice care, palliative medicine, #pallicovid to retrieve related content. Two studies^58 59^ retrieved advance-directives-related information by employing keywords for example #living will, #medical directive, #advancecareplan and #goals of care. The other keywords used in developing search filters are listed in table 2.

When applying the search filter to retrieve and collect data we identified three tools among existing studies: official data collection channels (eg, Twitter application programming interface (API)), third-party data collection tools (eg, Symplur Signals and TopsyPro), and manual collection. Three studies^13 53 59^ used the official data collection channel—Twitter API to collect Twitter data. Twitter API is designed for programmatic access to Twitter’s real-time and historical data. To use Twitter API, academic researchers have to apply for access permission. Three studies^15 44 45^ used third-party data collection tools like Symplur Signals^44 45^ and TopsyPro^15^ to access Twitter data. They are commercial social media analytics platforms to extract data from Twitter. Ten studies^12 46 49–52 54 55 57 58^ manually downloaded data from social media platforms.

Assessing the search filter is defined as validating the relevance of collected social media data to the research topic. Although the search filter was applied to screen out the collected data it did contain some irrelevant information. For instance, when we used the term ‘comfort care’ as a search term or synonym to retrieve tweets related to palliative and end-of-life care on Twitter we inadvertently identified a tweet describing the ‘Comfort Care’ brand of toilet paper which was irrelevant to our study. Therefore, it was necessary to assess the relevance of the collected data before analysis. A total of 1 520 329 posts, 325 videos and 33 online articles related to palliative and end-of-life care from 2008 to 2022 were collected in secondary analysis studies. Among them, 2056 tweets, 325 videos and 33 online articles, represented in 10 studies,^12 46 49–52 54 55 57 58^ were included for analysis after manually assessing whether it is related to palliative and end-of-life care or not. One study 13 employed a machine learning algorithm to identify and remove irrelevant tweets from the collected tweets but did not report further assessment for the rest of tweets. The remaining data in another five studies^15 44 45 53 59^ were included for analysis without reporting data assessment in their studies.

## Discussion

This systematic review aimed to identify and examine the use of social media in palliative and end-of-life care research by appraising the ethical and data collection issues associated with its uses and applications. Our review identified an increasing academic interest in using social media as a research tool in palliative and end-of-life care research. Specifically, our review highlighted three applications of social media as a secondary data source in palliative and end-of-life care research which included exploring the quality and features of online resources, engaging stakeholders to understand their experiences or thoughts and investigating surveillance on the frequency, trends and features of public conversation. We also identified that social media has been used as a platform for information sharing in palliative and end-of-life care research specifically to deliver the intervention, enhance recruitment opportunities and for promotion, education and training.

Of note, we identified how ethical issues that were considered and managed in social media studies were inconsistent. Most researchers reported research using social media data to be retrospective so ethical approval was often ignored or waived. However, when researchers attempted to obtain primary data through social media platforms, for example, by recruitment, ethical approval and participant consent were commonly required. The summary of data collection approaches revealed that a wide range of social media content related to palliative and end-of-life care was retrieved and analysed, however, data quality of completeness and accuracy still lacks validation.

### The use of social media in palliative and end-of-life care research

Casañas i Comabella and Wanat ’s review in 2015 emphasised the potential of social media in palliative care research recruitment to collect primary data.^6^ However, our review identified that social media use also extended to secondary data analysis (16 out of 21 included studies) to understand online discussion and resources. Given the data collection difficulties in palliative and end-of-life care research,^62^ it is not surprising that social media data have often acted as one complementary data resource in palliative care research. Social media data could also be seen as a new vehicle that provides agency for potentially vulnerable palliative care research participants with fragile physical and mental issues^22^ to share their views on their terms, in their time and when they feel able to do so.

The growing use of social media secondary data in palliative and end-of-life care may also be partly explained by rapid advancements in natural language processing technologies.^63^ These technologies allow researchers to extract more valuable information from unstructured social media data with higher efficiency. They have enabled palliative and end-of-life care researchers to identify different palliative and end-of-life care stakeholders,^45^ understand public sentiment,^15^ extract demographic features^13^ and conduct large-scale content analysis.^14^ Additionally, more research tasks can be addressed by using natural language processing techniques. For example, several studies have made use of natural language processing technologies as applied to social media data to examine healthcare performance,^64^ predict mental health states^65^ and identify patient-reported symptoms.^66^ These examples may offer those working in palliative care ways to examine the quality and satisfaction associated with palliative and end-of-life care services performance or identify issues associated with patients’ mental or physical symptoms. However, opportunities also come with challenges and the computer-assisted natural language processing technology poses new concerns about the accuracy and robustness of the results due to the ‘black-box’ analysis process.^67^

### Ethical implications for using social media in palliative and end-of-life care research

Attempts have been made in 2014 to construct ethical guidelines relevant to the use of social media in palliative care research.^68^ The ethical guidelines suggested (1) Internet discussions should be considered private and consent should be obtained for those who shared in for subsequent research; (2) A text-based analytical approach to social media data is not considered an appropriate method in the palliative research; (3) The use of historical text is problematic and not encouraged.^68^ Our review identified that in a number of instances, the way social media is currently being employed in palliative care research is not consistent with this guidance. Only 5 out of 21^10 47–49 61^ studies obtained participants’ consent when using social media. When it comes to using social media historical data, the situation has the potential to become complex; only 1 out of 16 studies^49^ obtained consent before collecting posts or other types of social media data. Moreover, most qualitative or mixed-methods studies used historical text-based data analysis. This was not to denigrate how social media was being used in palliative and end-of-life care research, but it suggests the field of inquiry has progressed since this guidance was first conceived.^68^

It is not explicitly stated in Twitter and Facebook’s privacy policies^69 70^ that access to historical data for research purposes requires additional consent from the users. However, implied consent should not be considered a default solution when it comes to public health research,^71^ especially for palliative and end-of-life care research.^68^ General Data Protection Regulation (GDPR) declares that the protection of natural persons in relation to the processing of personal data is a fundamental right.^72^ Social media data collection should be compliant with this regulation since it retrieves large amount of personal data. Some patients may share negative feelings at the end of life on social media, and may not want this to be known by their families or friends.^73^ Previous studies reviewing patients’ views indicated fears that patients may have when their sensitive, personal health data are used for the secondary purpose of research.^74 75^ Patients may worry about the confidentiality and anonymity of their personal data if it is used for research, adding more burden to their already fragile psychological condition. Therefore, obtaining consent from participants should be actively encouraged when appropriate. Nevertheless, big data research based on social media makes obtaining consent from every participant manifestly impracticable. Even so, it is necessary to assure public privacy and anonymity in all possible ways. Some possible mitigation strategies including data minimisation (ie, only collecting and storing data necessary to accomplish research objectives) and pseudonymisation have been suggested.^76 77^ Pseudonymisation is not yet available in the big data studies included in this literature review. General Data Protection Regulation (GDPR) states in recital 26 that, ‘The principles of data protection should therefore not apply to anonymous information, namely information which does not relate to an identified or identifiable natural person or to personal data rendered anonymous in such a manner that the data subject is not or no longer identifiable’. Therefore, it is especially important for text-based qualitative or mix-method studies where there is greater potential for processing identifiable information but it may also apply to quantitative studies.

Given our review identified ethical considerations associated with the use of social media varied, we suggest more flexible and proportionate ethical review criteria should be adopted depending on the application scenario. If palliative care researchers want to collect primary data through social media, including using social media to recruit, promote or deliver interventions, then obtaining ethical approval and participant consent must be required. If researchers want to access historical social media data to conduct secondary analysis, implied consent represents a challenge. This is the case where researchers are attempting to understand individual patient end-of-life trajectories or preferences or caregiver experiences where consent for their views is preferable. As a consequence of the evolving nature of social media, it is currently difficult to insist on a single ethical prescription for the application of social media in palliative and end-of-life care research. As the British Psychological Society Ethics Guidelines for Internet-Mediated Research pointed out, ‘Certain ethics principles may be more or less salient in different types of research design, and the procedures researchers put in place should be proportional to the likely risk to participants and researchers’.^78^

### Social media data collection and verification in palliative and end-of-life care research

Data collection and verification play a vital role in enhancing data accuracy and completeness. This is particularly important given the potentially large volume of social media data that can be interrogated since vagaries or idiosyncrasies in data collection can become magnified at scale.^79^

We identified among our included studies that developing and accessing search filters during the data collection process were sometimes not present or standardised. Specifically, we identified that only some studies (11 out of 16 studies) reported the step of assessing the search filter. This means some studies may not validate the retrieved data before analysis, especially for big data studies.^15 45 53 59^ It is risky to conduct research using data obtained solely through keywords on social media platforms without additional validation. Due to the complexity of the social media context, the search keywords are likely to retrieve irrelevant information. A commonly employed social media data collection framework^25^ has incorporated data validation as an essential element. In our review, we also emphatically recommend incorporating data validation into the data collection process in palliative and end-of-life care social media research. Even for big data studies, data validation should be conducted in a sample subset.

In addition, we also observed that when developing the search filter, it may be challenging to include all variants of one concept as search keywords. Sometimes the selected search keywords may be too technical that they are rarely used when describing palliative and end-of-life care by the public. Individuals tend to communicate more informally and colloquially on social media than they do in academic contexts.^25^ Snowball sampling may be a possible solution to combat the real-time updating of the internet which starts with retrieving a sample of tweets with ‘seed’ keywords and then identifying new keywords until no new keywords are found when repeating this process.^13^

We found the existing studies focused principally on English platforms with little attention being paid to non-English-speaking countries and regions. Access to palliative care is currently grossly inequitable between high-income and low-income counties.^80^ Moreover, the provision of palliative and end-of-life care services has distinct characteristics in different regions. Future studies should therefore attempt to use and explore palliative and end-of-life care content on non-English platforms.

### Strengths and limitations

To our knowledge, this is the first review to conduct a thorough systematic search of the available literature concerning social media uses in palliative and end-of-life care research. However, several limitations must be acknowledged. While we endeavoured to minimise language bias by conducting searches across various databases without imposing language restrictions, it is plausible that some non-English studies may have been inadvertently excluded due to their sole publication in local academic databases. Although we incorporated the Chinese academic database CNKI to encompass Chinese studies, some studies conducted in other non-English languages (eg, Japanese) and using local social media platforms (eg, LINE) may have been overlooked. Despite employing an extensive search strategy encompassing terms associated with social media, some studies that may have employed social media platforms might not have captured using our search terms. Given the rapid evolution of social media, it is challenging to enumerate the names of all social media platforms. A further limitation is our use of the QualSyst tool for quality assessment, which is not tailored for social media research. The absence of dedicated quality assessment tools for social media research highlights the need for future development in this field.

## Conclusion

Social media with real-time user-generated content and active user interaction opens up more possibilities for healthcare research. Researchers in palliative and end-of-life care have begun to explore the use of social media as an effective research tool to increase public knowledge and improve patients’ and caregivers’ quality of life. Our review identified an increasing interest in this field and summarised six applications of social media in palliative and end-of-life care research. To inform palliative and end-of-life care researchers who want to engage social media in their research, we also noticed and synthesised ethical considerations and data collection approaches of social media research. We identified inconsistent ethical handling and non-standardised data collection approaches among existing studies indicating potential risks in ethics and data quality. We have developed evidence-based recommendations to ensure the best possible ethical and data quality assurance of palliative social media research, including flexible ethical review criteria, ethical risk mitigation strategies, data validation and non-English platform research. Overall, this is a promising and fast-growing field, but continued efforts from the cross-discipline field (computer science, palliative care, media and communication) are needed to make further standardisation in terms of ethics and data quality.

## Data Availability

All data relevant to the study are included in the article or uploaded as online supplemental information.
