# Supplementary material for: Social media for palliative and end-of-life care research: a systematic review
Source: BMJ Support Palliat Care. 2024 Apr 9;14(2):149–62. doi: 10.1136/spcare-2023-004579 (PMC11103321; doi:10.1136/spcare-2023-004579)
Supplement: Supplementary data [file spcare-2023-004579supp001.pdf]

Supplemental file 1. Search strategy June 2020-December 2022

Ovid (including Embase, Medline, PsycInfo, Global Health) 2022-12-30

#1	(social media or social web or social network or web2 or web-based or twitter or tweet* or youtube or linkedin or instagram or reddit or weibo or wechat or online forum* or online community or pinterest or tumblr or tiktok or Patientslikeme or blog).mp.
#2	"web 2.0".mp.
#3	(palliative* or hospice or end of life or EoL* or PEoL* or terminal care or terminal ill* or advance care or marie curie nurse or macmillan nurse or comfort care or supportive care or bereavement care or respite care or pain management or symptom management or pain control).mp.
#4	1 or 2
#5	3 and 4

'multi-purpose' (.mp.) fields for: ti,bt,ab,ot,nm,hw,fx,kf,ox,px,rx,ui,sy,ux,mx.

Web of Science Core Colletion 2022-12-30

#1	((TS=(social media or social web or social network or "web 2.0" or (web2 or web-based) or (twitter or tweet* or youtube or linkedin or instagram or reddit or weibo or wechat or online forum* or online community or pinterest or tumblr or tiktok or Patientslikeme or blog))) AND TS=((palliative* or hospice or end of life or EoL* or PEoL* or terminal care or terminal ill* or advance care or marie curie nurse or macmillan nurse or comfort care or supportive care or bereavement care or respite care or pain management or symptom management or pain control)))

"Topic" (TS) fields for: Title, Abstract, Author Keywords, Keywords Plus.

CNKI 2022-12-30

#1	(TI,AB=(社交媒体+社交网络+微博+微信+推特+抖音+博客)*(姑息治疗+安宁疗护+舒缓医疗+缓和医疗+和缓医疗+临终关怀+姑息医学+安宁医疗+舒缓医学+安宁病房+预立医疗+预先医疗+医学预嘱+疼痛管理+疼痛控制+症状管理))

OpenGrey 2020-06-09 (through website)

#1	((social media or social web or social network or "web 2.0" or (web2 or web-based) or (twitter or tweet* or youtube or linkedin or instagram or reddit or weibo or wechat or online forum* or online community or pinterest or tumblr or tiktok or Patientslikeme or blog)) AND (palliative* or hospice or end of life or EoL* or PEoL* or terminal care or terminal ill* or advance care or marie curie nurse or macmillan nurse or comfort care or supportive care or

	bereavement care or respite care or pain management or symptom management or pain control)

OpenGrey 2022-12-30 (through MySQL database because the website is non-existent)

#1	SELECT * FROM item WHERE (title LIKE '%social media%' OR title LIKE '%social web%' OR title LIKE '%social network%' OR title LIKE '%web 2.0%' OR title LIKE '%web2%' OR title LIKE '%web-based%' OR title LIKE '%twitter%' OR title LIKE '%tweet%' OR title LIKE '%youtube%' OR title LIKE '% linkedin%' OR title LIKE '%instagram%' OR title LIKE '%reddit%' OR title LIKE '%weibo%' OR title LIKE '%wechat%' OR title LIKE '%online forum%' OR title LIKE '%online community%' OR title LIKE '%pinterest%' OR title LIKE '%tumblr%' OR title LIKE '%tiktok%' OR title LIKE '%patientslikeme%' OR title LIKE '%blog%') AND (title LIKE '% palliative%' OR title LIKE '%hospice%' OR title LIKE '%end of life%' OR title LIKE '%EoL%' OR title LIKE '%terminal care%' OR title LIKE '%terminal ill%' OR title LIKE '%advance care%' OR title LIKE '%marie curie nurse%' OR title LIKE '%macmillan nurse%' OR title LIKE '%comfort care%' OR title LIKE '%supportive care%' OR title LIKE '%bereavement care%' OR title LIKE '%respite care%' OR title LIKE '%pain management%' OR title LIKE '%symptom management%' OR title LIKE '%pain control%');

CareSearch 2022-12-30

#1	(((((((((((((((((“social media”[Abstract]) OR “social web”[Abstract]) OR “social network”[Abstract]) OR “web 2.0”[Abstract]) OR web2[Abstract]) OR web-based[Abstract]) OR twitter[Abstract]) OR tweet[Abstract]) OR youtube[Abstract]) OR linkedin[Abstract]) OR instagram[Abstract]) OR reddit[Abstract]) OR weibo[Abstract]) OR wechat[Abstract]) OR “online forum”[Abstract]) OR “online community”[Abstract]) OR pinterest[Abstract]) OR tumblr[Abstract]) OR tiktok[Abstract]) OR Patientslikeme[Abstract]) OR blog[Abstract], Type: Research Studies
